# Genomic Characterization of Clinical *Staphylococcus aureus* Isolates from Ecuador Reveals Diverse Lineages and Genomic Similarity Among Sequence Type 2625 Isolates

**DOI:** 10.3390/ijms27209135

**Published:** 2026-10-15

**Authors:** Ariana Vinueza, Miroslava Anna Šefcová, Isaías Mejía Limones, Laura Bejarano, Renáta Szaboová, Róbert Herich, Gabriela Irene Andrade Mena, Anahí Rosero-Tixicuro, Pedro Barba, Marco Larrea-Álvarez

**Affiliations:** 1 Carrera de Medicina, Facultad de Ciencias de la Salud, Universidad Espíritu Santo, Samborondón 092301, Ecuador; 2 Department of Biology and Physiology, University of Veterinary Medicine and Pharmacy in Košice, 04181 Košice, Slovakia; 3 Department of Morphological Disciplines, University of Veterinary Medicine and Pharmacy in Košice, 04181 Košice, Slovakia; 4 Laboratorio Clínico, Hospital General San Vicente de Paúl, Ibarra 100150, Ecuador; 5 Carrera de Biotecnología, Facultad de Ingeniería en Ciencias Agropecuarias y Ambientales, Universidad Técnica del Norte, Ibarra 100150, Ecuadorpmbarba@utn.edu.ec (P.B.)

**Keywords:** *Staphylococcus aureus*, antimicrobial resistance, MRSA, whole-genome sequencing, genomic epidemiology, phylogenomics, virulence, ST8, ST30, ST72, ST2625

## Abstract

*Staphylococcus aureus* is characterized by substantial genomic diversity, with lineages differing in antimicrobial resistance, virulence-associated determinants, and accessory genetic content. However, genomic data from Ecuador remain comparatively limited. Here, we characterized five clinical *S. aureus* isolates recovered in Ibarra, Ecuador, using phenotypic antimicrobial susceptibility testing and whole-genome sequencing, and compared them with publicly available Ecuadorian genomes. The isolates represented four sequence types (ST8, ST30, ST72, and ST2625), including four MRSA isolates and one MSSA isolate. Whole-genome analysis identified differences in antimicrobial resistance determinants, virulence-associated genes, and replicons, while phenotypic susceptibility profiles generally corresponded with the detected resistance traits. The Ecuadorian collection showed a diverse population structure, with ST8 predominating. Placement within globally circulating lineages showed that the ST8 isolates clustered with USA300-LV (CA12), the ST30 isolate with G2-Sg2-Pr, and the ST72 isolate with clade B. A focused phylogenomic analysis of ST2625 showed limited genomic divergence among Ecuadorian and international isolates, with broadly similar resistance and virulence-associated profiles. These findings add genomic data from northern Ecuador and illustrate variation in the genetic composition of circulating *S. aureus* lineages. Continued genomic surveillance across additional geographic settings may help clarify the distribution and diversity of *S. aureus* in the country.

## 1. Introduction

*S. aureus* is a major human pathogen and a common colonizer of the skin and anterior nares [1,2]. It is able to cause a broad spectrum of infections, ranging from skin and soft-tissue infections to invasive diseases such as bacteremia, osteomyelitis, and pneumonia [3,4]. The genetic plasticity of *S. aureus* enables the acquisition and maintenance of antimicrobial resistance (AMR) and virulence determinants, which facilitate its dissemination into hospital and community settings [5]. From a clinical perspective, its population has been distinguished as methicillin-resistant (MRSA) or methicillin-susceptible (MSSA). While the former has received substantial attention because of its antimicrobial resistance and epidemiological importance, the latter remains an important cause of human infection and is represented by multiple successful lineages [6,7]. Thus, characterization of both MRSA and MSSA population structures is crucial for understanding the distribution of clinically relevant genetic traits as well as their circulation across different settings.

Antimicrobial resistance is a major feature of *S. aureus* epidemiology, with β-lactam resistance remaining particularly important because of its longstanding use for the treatment of staphylococcal infections [8,9]. Penicillin resistance is commonly mediated by the *blaZ–blaR1–blaI* system, in which *blaZ* encodes a β-lactamase, while the regulatory genes contribute to its expression [10]. The acquisition of *mecA* further expanded the clinical importance of resistant *S. aureus* by enabling methicillin resistance through production of PBP2a, a penicillin-binding protein with reduced affinity for β-lactams [6,11]. This gene is carried within the staphylococcal cassette chromosome *mec* (SCCmec), a mobile genetic element that carries *mecA* and occurs in multiple structural types associated with different MRSA lineages [12]. In addition to β-lactams, *S. aureus* can acquire resistance determinants affecting macrolides, tetracyclines, aminoglycosides, and trimethoprim [13,14,15,16].

While phenotypic antimicrobial susceptibility testing provides information on expressed resistance, it must be complemented by the identification of the underlying genetic determinants [17,18]. Whole-genome sequencing (WGS) not only allows the characterization of sequence type, antimicrobial resistance and virulence genes, SCCmec elements, plasmids, and other accessory genetic features, but also supports assessment of phylogenetic relationships among isolates [17,18]. This combination can help describe population structure together with variation in the accessory genome [19,20,21,22]. Importantly, the presence of a resistance determinant does not necessarily correspond to phenotypic resistance, as factors such as gene expression, genetic context, and allele functionality may influence the outcomes [23,24]. Therefore, integrating WGS with phenotypic susceptibility testing provides complementary information that enables a more comprehensive assessment of resistance profiles and genomic diversity.

The population structure of *S. aureus* comprises numerous multilocus sequence typing (MLST) lineages and clonal complexes, some of which have been identified across multiple geographic and clinical settings [25,26,27], including ST8, ST5, ST30, ST22, and ST72. These lineages may differ in the carriage of antimicrobial resistance determinants, SCCmec elements, plasmids, and other accessory factors [28,29,30,31,32]. Virulence represents an additional component of this genomic diversity. Many lineages carry conserved genes involved in adhesion, biofilm formation, immune evasion, and iron acquisition, which contribute to colonization and persistence [33,34,35]. In contrast, toxin-associated determinants such as Panton–Valentine leukocidin (PVL) and toxic shock syndrome toxin-1 (TSST-1) can vary among lineages [36,37,38,39]. Arguably, the characterization of these lineage-associated differences can provide a broader view of *S. aureus* population structure and the distribution of key genetic traits.

Across Latin America, *S. aureus* populations include multiple internationally distributed lineages, although the extent of genomic surveillance varies among countries [40,41]. In Ecuador, previous studies have identified a diverse population with several sequence types including ST8, ST5, ST30, and ST22 [40,41,42]. However, both the isolates reported in these studies and the publicly available Ecuadorian genomes originate primarily from Quito, so this geographic distribution should be considered when interpreting the available genomic data. Characterization of clinical isolates from different regions could contribute to understanding variation not only in lineage distribution, but also in crucial genomic features. Moreover, the potential identification of several less frequently reported lineages supports the value of expanding characterization to different cities [40,43]. For instance, ST2625 remains relatively underrepresented in publicly available genomic datasets, with genomes reported from countries including Italy and Spain [44,45,46]. Undoubtedly, data from additional geographic settings may provide further insight into the diversity and evolution of this sequence type.

In this context, the goal of this study was to characterize five clinical *S. aureus* isolates from Ecuador using phenotypic antimicrobial susceptibility testing, along with WGS, and compare them with reference genomes and publicly available Ecuadorian isolates. We examined antimicrobial resistance determinants, virulence-associated genes, replicon content, population structure, and phylogenetic relationships, with particular attention to ST2625.

## 2. Results

### 2.1. Clinical Isolates and Genomic Characteristics

The five isolates were identified as *S. aureus* based on their characteristic microbiological profile. Three isolates (SAT1, SAT2, and SAP1) were recovered from community-associated wound infections, whereas SAI1 (cerebrospinal fluid, intensive care unit) and SAS1 (surgical wound) were hospital-associated. Antimicrobial susceptibility testing revealed that all isolates were resistant to penicillin G and susceptible to ciprofloxacin, trimethoprim-sulfamethoxazole, and linezolid (Table 1) (Supplementary Results: Raw Data). Four isolates (SAT2, SAP1, SAI1, and SAS1) were resistant to cefoxitin, indicating a methicillin-resistant phenotype, while SAT1 remained susceptible. Resistance to azithromycin and erythromycin was detected only in SAT1 and SAI1. Tetracycline and gentamicin resistance was confined to the two hospital-associated isolates (SAI1 and SAS1), whereas the community isolates were susceptible to both agents.

Genome sizes ranged from 2.79 to 2.85 Mb, with GC contents of approximately 32.7%, encoded between 2587 and 2695 coding sequences, and showed high assembly quality (completeness ≥ 99.94%, contamination ≤ 0.12%, N50 94,883–216,386 bp, sequencing depth 14×–32×) (Supplementary Results: Raw Data). All five isolates showed ≥97.5% ANI to NCTC 8325 and ATCC 51811 (Supplementary Results: Table S1). MLST analysis identified four STs, with ST8 represented by two community-associated isolates (SAT2 and SAP1), with the others being ST30 (SAT1), ST72 (SAI1), and the uncommon ST2625 (SAS1). Four isolates, belonging to ST8, ST72, and ST2625, harbored *mecA*, consistent with their methicillin-resistant phenotype, whereas the ST30 isolate lacked this sequence. SCCmec type IV was identified in three isolates, while the ST8 isolate SAT2 carried an untypeable element. The resistome reflected the phenotypic susceptibility profiles, with β-lactam resistance genes present in all isolates, whereas additional determinants conferring resistance to aminoglycosides, macrolides, tetracyclines, folate antagonists, bleomycin, and streptothricin were also detected (Table 2).

**Figure 1:**
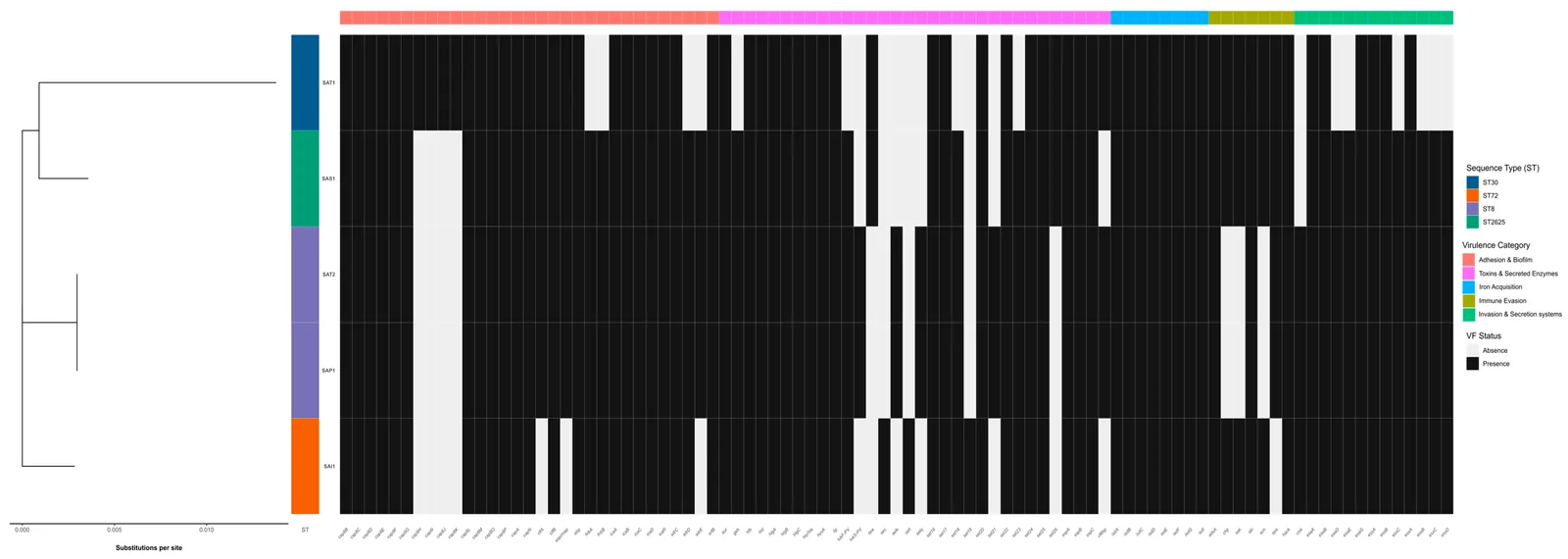
Virulence gene profile of the five clinical *S. aureus* isolates. Maximum-likelihood phylogeny (left) based on core-genome substitutions per site, with sequence types indicated by colored bars (ST30, ST72, ST8, ST2625). The heatmap shows the presence (black) or absence (white) of virulence-associated genes identified through the VFDB, grouped by functional category. Gene names are listed below the heatmap.

Replicon analysis identified one to three elements per isolate, with ST2625 harboring the largest content. Mobile genetic element screening identified four to six insertion sequences per isolate, with Tn554 in the ST30 isolate and IS431mec in the ST8 (SAP1) and ST2625 isolates. Two intact prophages were identified per isolate, with *S. aureus* phage 11 as the most common match and *S. aureus* PT1028 detected only in the ST30 isolate. The two ST8 isolates shared nearly identical intact prophage regions (Table 2). Virulence profiling revealed a highly conserved core virulome, including genes associated with adhesion, biofilm formation, iron acquisition, immune evasion, and the type VII secretion system. Nevertheless, differences were observed in toxin-associated genes, with PVL genes (*lukF-PV* and *lukS-PV*) detected in the ST8 isolates, whereas enterotoxin genes varied among sequence types (Figure 1).

### 2.2. Phylogenetic Analysis of Ecuadorian S. aureus Isolates

**Figure 2:**
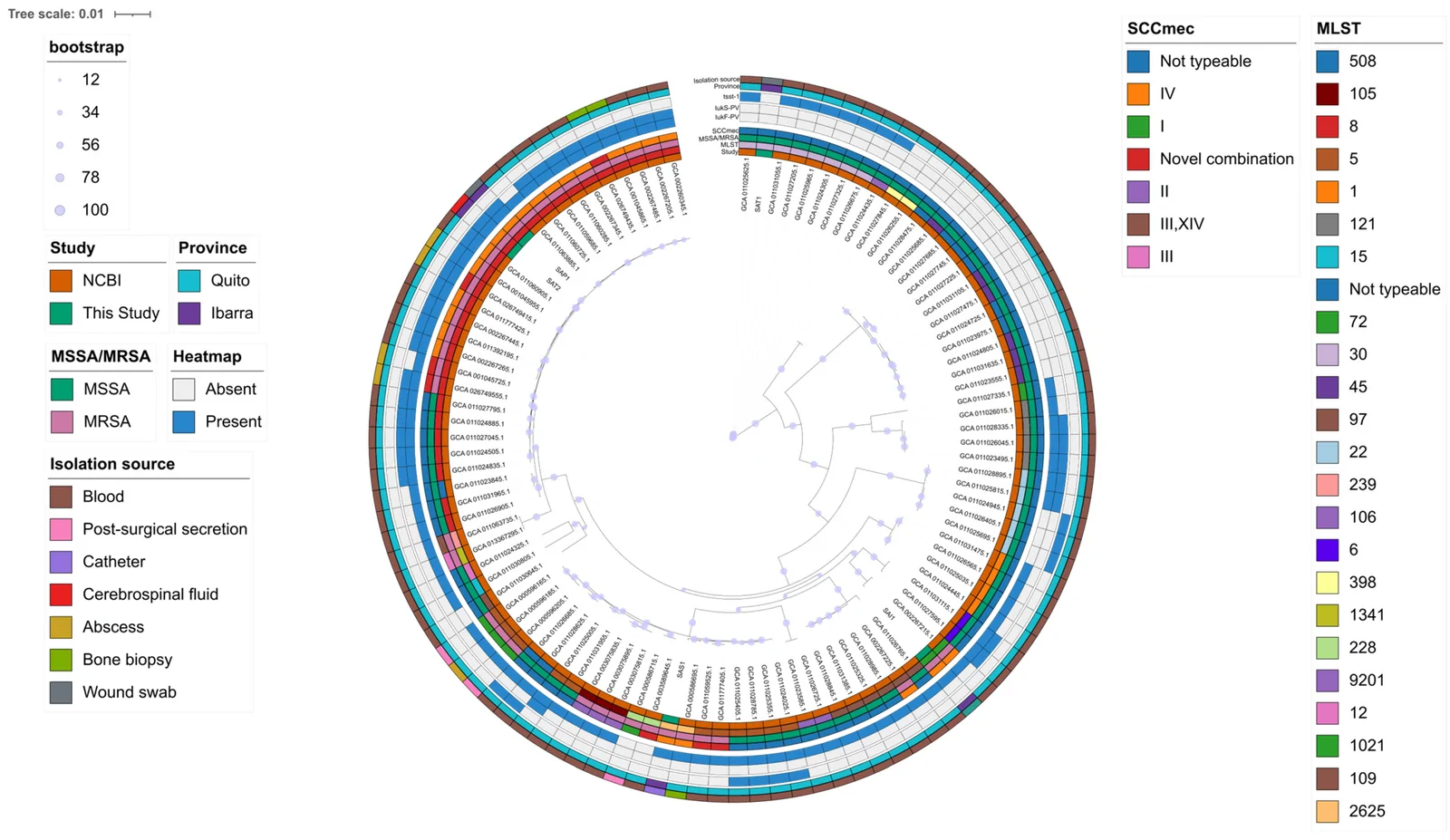
Phylogenomic analysis of *S. aureus* isolates. Maximum-likelihood phylogenomic tree inferred from the core-genome alignment of isolates included in this study together with publicly available Ecuadorian genomes retrieved from NCBI. The concentric annotation rings (from inner to outer) represent isolate origin (this study or NCBI), geographic location (province), methicillin susceptibility phenotype (MSSA/MRSA), isolation source, presence/absence of AMR genes, SCCmec type, and MLST. Node sizes are proportional to bootstrap support values. Branch lengths are scaled according to the number of nucleotide substitutions per site.

Figure 2 presents the core-genome phylogeny of our five isolates together with 98 publicly available Ecuadorian genomes. The combined dataset comprised 103 sequences distributed across multiple STs, demonstrating substantial genomic diversity. ST8 was the predominant lineage (28/103, 27.2%), followed by ST5 (11/103, 10.7%), ST30 (8/103, 7.8%), ST45, ST97 (6/103, 5.8%), and ST22 (5/103, 4.9%), while the remaining genomes were assigned to less frequent lineages, including ST72 and the uncommon ST2625. Our isolates recovered from Ibarra broaden the geographic representation as the publicly available genomes all originated from Quito. Overall, 65 of them (63.1%) were classified as MSSA and 38 (36.9%) as MRSA. Among MRSA isolates, SCCmec type IV was the most common cassette, although types I, II, III, hybrid III/XIV, and untypeable SCCmec elements were also identified. Our genomes were distributed across four distinct lineages. SAT2 and SAP1 clustered within the predominant ST8, which consisted mainly of MRSA isolates (21/28) carrying SCCmec type IV or untypeable SCCmec elements. ST8 isolates were recovered primarily from bloodstream infections, although isolates from abscesses, bone biopsies, wound swabs, and cerebrospinal fluid were also represented. This lineage was characterized by the widespread presence of *lukF-PV* (100%) and frequent detection of *lukS-PV* (24/28), whereas *tsst-1* was absent. SAT1 grouped within the ST30 lineage, composed exclusively of MSSA isolates recovered predominantly from bloodstream infections. Unlike ST8, ST30 lacked both PVL genes but frequently harbored *tsst-1* (7/8 isolates). SAI1 clustered with the two-member ST72 lineage, whose isolates were MRSA carrying SCCmec type IV and recovered from blood and wound secretions. Both ST72 isolates encoded *lukF-PV* but lacked *lukS-PV* and *tsst-1*. Finally, SAS1 clustered with the only other Ecuadorian ST2625 genome. This rare lineage was composed exclusively of MRSA isolates carrying SCCmec type IV, recovered from catheter-associated infection and bone biopsies, and sharing the same toxin profile as ST72. The phylogeny demonstrates that the isolates generated in this study are representative of both common circulating Ecuadorian clones (ST8 and ST30) and rarely reported lineages (ST72 and ST2625).

**Figure 3:**
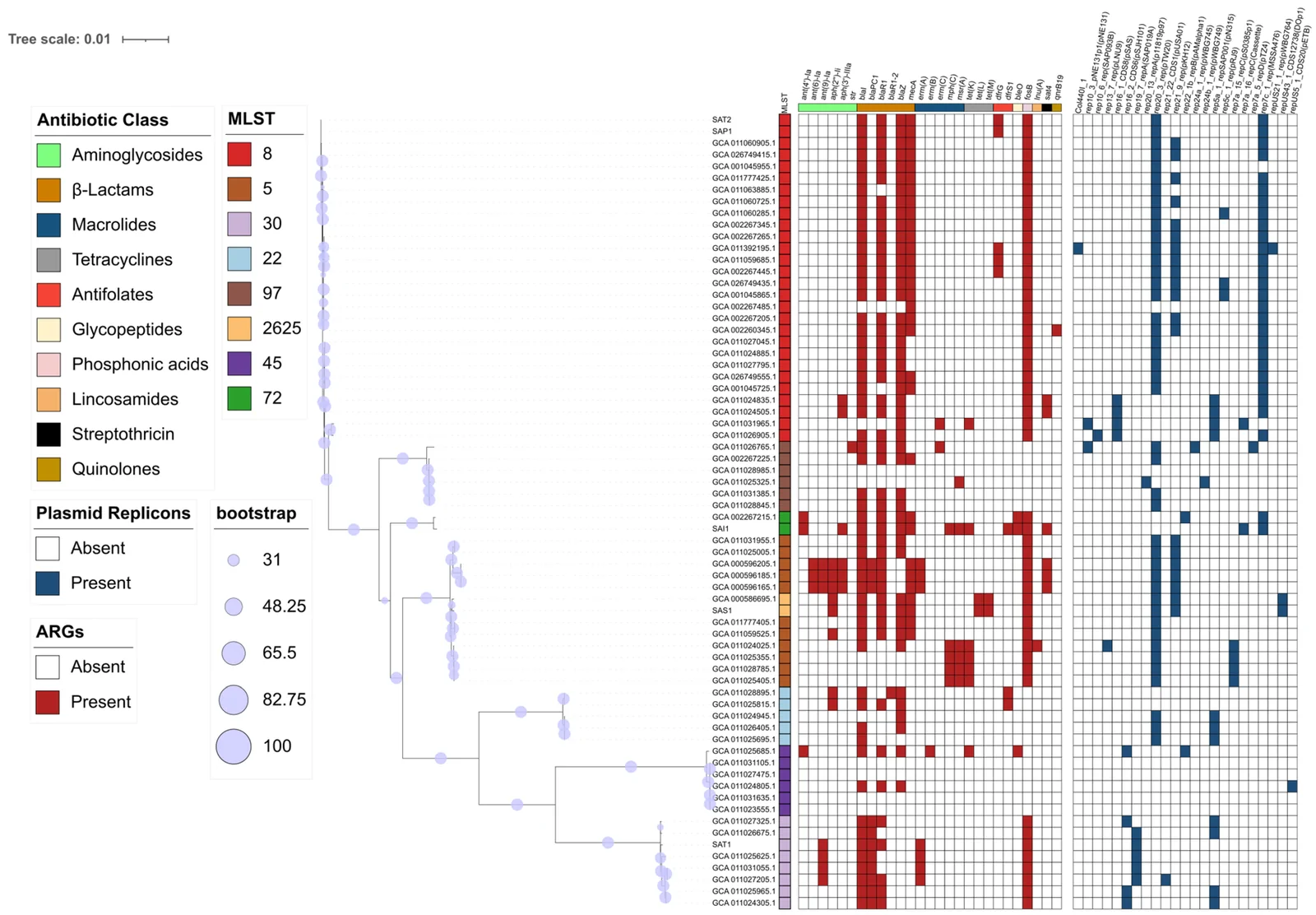
Distribution of antimicrobial resistance genes and replicons. Maximum-likelihood phylogenetic tree showing the distribution of AMR genes (left heatmap, red) and replicons (right heatmap, blue) among the eight most prevalent *S. aureus* STs identified in the Ecuadorian dataset. Isolates are grouped according to the core-genome phylogeny. The colored bar above the heatmap indicates the antimicrobial class associated with each resistance determinant, while the vertical annotation bar indicates the MLST. White cells indicate gene or replicon absence, whereas colored cells indicate presence. Node sizes are proportional to bootstrap support values, and branch lengths are scaled according to the number of nucleotide substitutions per site. ARG: antimicrobial resistance genes.

The phylogenetic relationships among the eight most common STs, as well as their corresponding AMR genes and replicons, are presented in Figure 3. Isolates clustered according to sequence type. The β-lactam resistance genes *blaI*, *blaR1*, and *blaZ* were the most frequently detected determinants and were present across nearly all lineages, whereas *mecA* was restricted to MRSA-associated STs, including ST8, ST72, ST5, ST22, ST2625, and a subset of ST97 isolates. Resistance traits for aminoglycosides, macrolides, tetracyclines, trimethoprim, bleomycin, and streptothricin showed variable distributions among sequence types. ST72 contained the broadest resistome, carrying genes associated with resistance to seven antimicrobial classes, whereas ST30 displayed the fewest acquired resistance genes. ST5 exhibited considerable heterogeneity, with isolates differing in aminoglycoside, tetracycline, and trimethoprim resistance genes, while ST45 and ST97 were characterized by relatively conserved resistance profiles. Replicons also varied among sequence types. Replicons related to pTW20 and MSSA476 predominated in ST8, whereas ST5 contained the greatest diversity of replicons. ST2625 carried three distinct replicons, including pTW20- and pKH12-related elements, while ST72 was characterized by MSSA476-related replicons. In general, the resistome and replicon profiles of the five characterized isolates were consistent with those of publicly available genomes belonging to the same sequence type.

**Figure 4:**
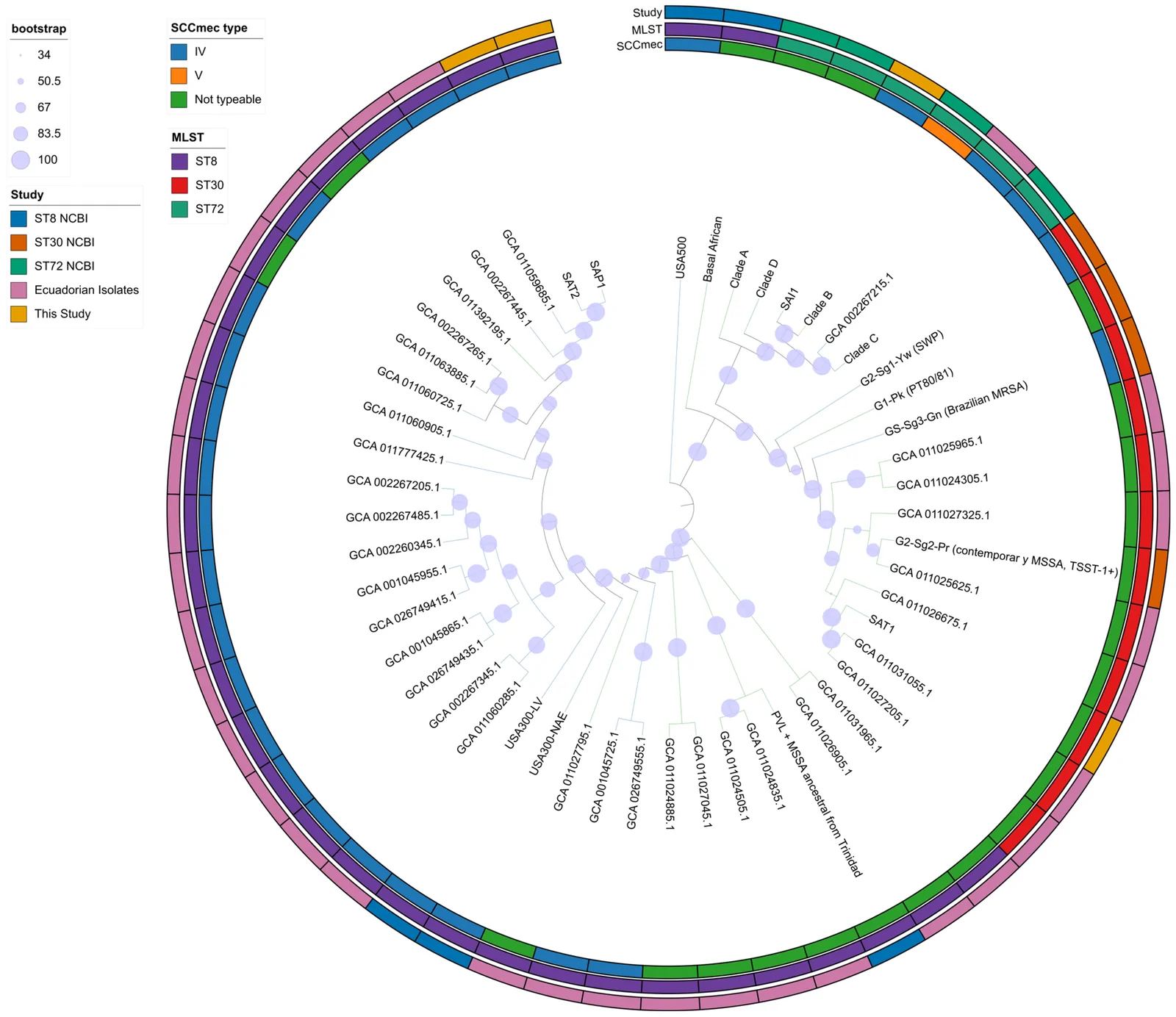
Phylogenetic tree of *S. aureus* isolates and reference genomes based on WGS data. The tree includes isolates from this study together with publicly available NCBI reference genomes, representing sequence types ST8, ST30, and ST72, and previously characterized Ecuadorian isolates. Branches are colored according to the corresponding phylogenetic grouping, while circular node symbols indicate bootstrap support, with symbol size proportional to bootstrap values. The outer annotation rings display the study origin, MLST sequence type, and SCCmec type (IV, V, or non-typeable) for each genome.

The five study isolates clustered by sequence type alongside their corresponding reference genomes within the phylogeny (Figure 4). SAT2 and SAP1 (ST8) clustered with the USA300-LV (Latin American) reference (CA12), separately from USA300-NAE (FPR3757) and USA500. Both isolates carried a complete COMER element (*merA*, *merB*, *merR*, *copZ*, *mco*) and lacked the ACME-specific *opp* operon. ANI was highest in USA300-LV (≥99.93%) and lower in USA300-NAE (≤99.92%) and USA500 (99.78%). Against Ecuadorian ST8 genomes, ANI ranged from 99.27% to 99.96%, with the highest values being 99.9% (Supplementary Results: Table S1). The two isolates differed by five core-SNPs. SAT1 (ST30) clustered with the G2-Sg2-Pr subgroup and did not carry *tsst-1* and PVL genes. Among the four ST30 subclade references, the highest ANI was for G2-Sg2-Pr (99.80%). Against Ecuadorian ST30 genomes, ANI ranged from 99.73% to 99.89% (Supplementary Results: Table S1). SAI1 clustered with the ST72 references and carried SCCmec type IV and PVL genes. ANI was highest for Clade B (99.94%) and lower (99.89%) for the only Ecuadorian ST72 genome available (GCA_002267215.1) (Supplementary Results: Table S1).

### 2.3. Phylogenetic Analysis of ST2625 Isolates

**Figure 5:**
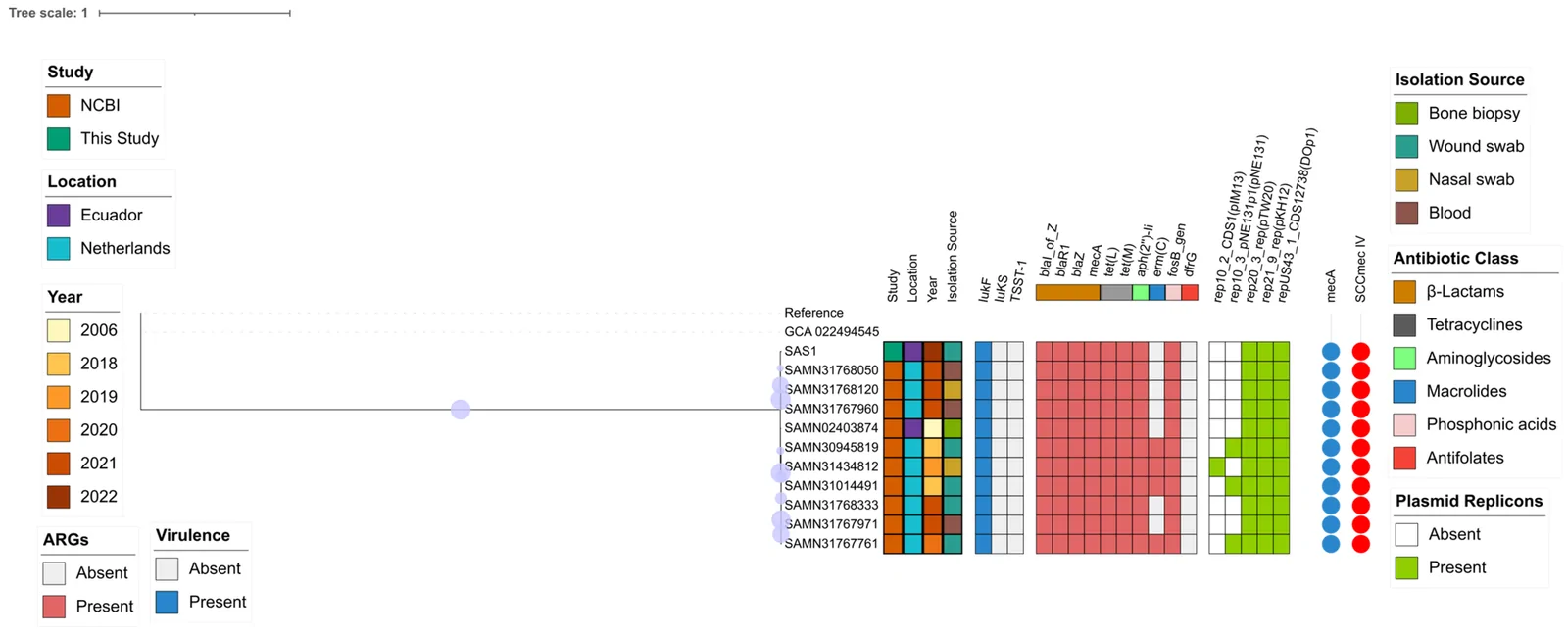
Comparative genomic characterization of *S. aureus* ST2625 isolates. Maximum-likelihood phylogenetic tree of ST2625 isolates from Ecuador and publicly available genomes. Metadata annotations include geographic location, year of isolation, isolation source, presence/absence of virulence genes, replicons, and antimicrobial classes represented by the detected resistance determinants. The adjacent heatmaps indicate the presence (colored) or absence (white) of virulence as well as antimicrobial resistance genes, and replicons. Bootstrap support is represented by node size. ARG: antimicrobial resistance genes.

The analysis revealed limited diversity within the lineage, with all isolates clustering closely despite differences in geographic origin and year of isolation (2006–2022) (Figure 5). The Ecuadorian isolates grouped within the same clade as the Dutch genomes, indicating a high degree of genomic similarity. ANI values ranged from 99.90% to 99.97%, with 99.92% for the Ecuadorian ST2625 genome.

Clinical sources included wounds (*n* = 6), blood (*n* = 3), nasal swabs (*n* = 2), and osteomyelitis (*n* = 1). All ST2625 isolates were classified as MRSA, carried *mecA*, and possessed SCCmec type IV. The AMR profile was highly comparable, with all genomes harboring *blaI*, *blaR1*, *blaZ*, *fosB*, *aph (2″)-Ii*, *tet (L)*, and *tet (M)*. In contrast, *erm (C)* was detected only in the Dutch isolates collected between 2018 and 2020 and was absent from both Ecuadorian genomes and the Dutch isolates recovered in 2021. None of the genomes carried *dfrG*. The virulence profile was similar across genomes. All isolates encoded *lukF-PV*, whereas *lukS-PV* and *tsst-1* were not detected. Likewise, the replicon heatmap showed a largely conserved replicon profile, with only minor variation among isolates. The SAS1 isolate shared the characteristic resistance and virulence profile of the ST2625 lineage, differing only in replicon content from a subset of Dutch genomes.

## 3. Discussion

Five clinical *S. aureus* isolates recovered from community- and hospital-associated infections were characterized by phenotypic antimicrobial susceptibility testing and WGS, revealing genetic diversity across four sequence types (ST8, ST30, ST72, and ST2625). Four isolates were classified as MRSA and one as MSSA, consistent with the detection of *mecA* and SCCmec elements. Comparison with 98 publicly available Ecuadorian genomes indicated that the study isolates included both prevalent and less frequently reported circulating lineages. Comparative genomic analyses identified differences in AMR determinants, virulence factors, replicon repertoires, and phylogenetic relationships. In addition, the ST2625 isolates exhibited limited genomic divergence from the few internationally available genomes.

Phenotypic susceptibility showed overall concordance with WGS predictions. Cefoxitin resistance corresponded to *mecA* carriage and the MSSA isolate lacked this determinant [47,48]; *blaZ* detection was consistent with penicillin G resistance [10,49]; *erm (A)* and *mph (C)*/*msr (A)* corresponded to the macrolide-resistant phenotypes of SAT1 and SAI1 [13,50]; tetracycline resistance coincided with *tet (K)* or *tet (L)*/*tet (M)*, and aminoglycoside-modifying enzymes were detected among gentamicin-resistant isolates [14,15,51]; and trimethoprim-sulfamethoxazole susceptibility was observed in isolates carrying *dfr* genes [16].

The phylogenetic analysis indicated that the Ecuadorian collection is genetically diverse, with isolates distributed across various STs. Similar polyclonal population structures have been reported in other Latin American countries, where several internationally recognized lineages co-circulate in both community and healthcare settings [40,41]. Although MSSA predominated in the collection, SCCmec type IV was the most frequently identified cassette among MRSA isolates. This observation is consistent with the widespread distribution of SCCmec IV among major MRSA clones, particularly ST8, which has been reported beyond its original association with community-associated MRSA [52,53,54]. ST8 was also the predominant type in our collection, followed by ST5, ST30, ST45, ST97, and ST22, consistent with the presence of several internationally distributed lineages reported in human infections worldwide [28,55,56]. Our analysis confirmed the presence of 12 STs previously reported in Ecuador (ST8, ST5, ST30, ST45, ST22, ST97, ST121, ST398, ST105, ST239, ST109, and ST15) [42], while additionally identifying 11 STs that, to our knowledge, have not been described in peer-reviewed studies from the country (ST72, ST2625, ST228, ST106, ST12, ST9201, ST1, ST508, ST1021, ST6, and ST1341). Several of these lineages, including ST1, ST12, ST228, and ST508, have been associated with clinically important infections in other geographic areas [57,58,59,60].

Consistent with previous reports from Ecuador, ST8 included predominantly MRSA isolates, although MSSA representatives have also been described [42]. Likewise, all ST30 isolates in our collection were MSSA, in agreement with a previous study from Quito that identified eight MSSA isolates and one MRSA isolate belonging to this lineage [42], which has been particularly associated with methicillin-susceptible cases in several settings [30]. ST72 was identified both in our collection and among publicly available Ecuadorian genomes. Although this lineage has historically been associated with East Asia, its detection in Ecuador is consistent with recent reports documenting ST72 in diverse geographic regions beyond Asia [40,43]. To our knowledge, ST72 has not been reported previously in a peer-reviewed publication from Ecuador, with only an undergraduate thesis documenting its presence in a hospital in Quito [61].

SAT2 and SAP1 clustered within the USA300-LV (Latin American) clade as defined by Strauß et al. [28], carried COMER but not the ACME-specific *opp* operon, and differed by only five core SNPs, suggesting that they represent a single locally circulating USA300-LV clone. This is consistent with the documented prevalence of the Latin American variant in South America. SAT1 grouped within the G2-Sg2-Pr subgroup described by Esteves et al. [30], the contemporary MSSA lineage that concentrates most TSST-1-positive strains; SAT1, however, did not carry this trait and was PVL-negative. SAI1 grouped with the ST72 references as defined by Zhou et al. [43], most closely with Clade B, described as a highly virulent clade carrying PVL in some isolates, consistent with the SCCmec type IV and PVL-positive profile of SAI1. Across all three lineages, ANI values between the study isolates and Ecuadorian genomes of the same sequence type were uniformly high, indicating that the study isolates are closely related to previously described Ecuadorian lineages rather than representing novel or divergent lineages.

The widespread distribution of the β-lactamase operon across major *S. aureus* clones aligns with the long-standing dissemination of β-lactamase-mediated penicillin resistance in both MSSA and MRSA populations, in which the *bla* regulatory system is commonly associated [62,63]. In contrast, *mecA* was detected exclusively among MRSA isolates belonging to ST8, ST5, ST22, ST72, and a subset of ST97 isolates, consistent with the distribution of SCCmec among major MRSA lineages reported globally [28,31,32,64,65]. Accessory resistance determinants for aminoglycosides, macrolides, tetracyclines, trimethoprim, bleomycin, and streptothricin varied among sequence types, which is compatible with the differences in accessory genome composition described in *S. aureus* [19,20]. Within the analyzed collection, ST72 showed a broad resistome, whereas ST30 contained comparatively few genes; ST5 showed heterogeneous resistance profiles and the greatest replicon diversity [29,66,67]. Replicon profiles also exhibited association patterns among the analyzed genomes, with pTW20- and MSSA476-related replicons predominating in ST8 [68,69], while ST5 displayed the greatest replicon diversity [29]. Similar lineage-associated patterns have been reported elsewhere and may contribute to differences in accessory genome composition and the distribution of antimicrobial resistance determinants [19,21,22].

The reported isolates shared a broadly conserved repertoire of virulence determinants involved in adhesion, biofilm formation, immune evasion, iron acquisition, and Type VII secretion, consistent with their roles in *S. aureus* colonization, adaptation, and persistence [33,34,35]. In contrast, toxin-associated genes showed greater variation [70,71]. PVL genes were primarily detected among ST8 isolates, aligning with reports describing an association between ST8 and PVL carriage [36,72]. However, the contribution of PVL to disease severity remains controversial. Large studies have found no association with mortality in bacteremia [37,73], whereas PVL has also been reported in association with severe and fatal necrotizing pneumonia [74,75]. Likewise, the predominance of *tsst-1* among ST30 isolates agrees with previous reports of frequent toxin carriage in this lineage [38,39]. This gene was not detected in the ST72 isolate; its absence has also been reported in this clone in previous studies [66,76]. Nevertheless, *tsst-1* has been identified in a specific MSSA subclade (clade D), suggesting that its distribution may vary within the lineage rather than being uniformly absent [43]. The ST2625 isolate carried a virulence profile broadly comparable to previously reported ST2625 strains, including PVL-positive (*lukF-PV*) strains, *tsst-1*-negative strains, Immune Evasion Cluster (IEC)-positive (*scn*) strains, and conserved determinants associated with adhesion, T7SS, immune evasion, and iron acquisition [44,45,46]. However, the clinical significance of these genomic profiles cannot be inferred from the present data, and their potential functional relevance warrants further investigation across different hosts and niches [21,77].

The identification of ST2625 adds to the currently recognized diversity of *S. aureus* reported in the country. Given the limited number of ST2625 genomes described worldwide, its detection supports the value of continued genomic surveillance to better understand the distribution and genomic diversity of this infrequently reported lineage [44,45,46]. To our knowledge, this represents the first genomic characterization of ST2625 reported from Ecuador in the peer-reviewed literature. Following its initial description during an Italian neonatal intensive care unit outbreak [44], only two ST2625 isolates have been described in Spain, both from a tetracycline-resistant MRSA collection [45], along with one pediatric CA-MRSA case [46]. The Ecuadorian and Dutch ST2625 genomes analyzed here shared several genomic features, including *mecA*, the β-lactamase operon (*blaI–blaR1–blaZ*), *tet (L)*, *tet (M)*, aminoglycoside resistance determinants, SCCmec type IV, and PVL-associated genes, while *tsst-1* was absent. The main difference observed in the present collection was the detection of *erm (C)* in a subset of Dutch isolates, indicating some variation in the accessory resistome. Nevertheless, the small number of ST2625 genomes warrants cautious interpretation, as the observed genomic similarity may reflect limited diversification, recent dissemination, or sampling biases, which can influence population genomic analyses in *S. aureus* [78,79]. Expanding genomic surveillance to include ST2625 isolates from additional geographic regions will help determine whether these features are maintained in larger collections and further clarify the lineage’s genetic diversity and evolutionary relationships, as illustrated for other lineages such as ST188 and ST59 [22,80].

A strength of this study is the integration of phenotypic and genomic data with comparative phylogenomic analyses of Ecuadorian *S. aureus* isolates, including characterization of the uncommon ST2625 lineage. Several limitations should be considered. The isolates were not selected using predefined criteria but represented the non-duplicate Gram-positive cocci available from the hospital collection. Together with the small number of retrospective isolates from a single hospital, the absence of long-read sequencing, and the lack of functional validation, this limits the generalizability of the findings to the broader Ecuadorian *S. aureus* population. Wider genomic surveillance incorporating isolates from multiple healthcare settings and geographic regions, together with clinical, phenotypic, and WGS data, would help better characterize the distribution and evolution of *S. aureus* lineages and AMR in Ecuador.

## 4. Materials and Methods

### 4.1. Bacterial Isolates

The five isolates included in this study were selected from the routine clinical microbiology collection of Hospital San Vicente de Paúl, Ibarra, Ecuador. Five non-duplicate isolates, recovered during March 2021 from distinct clinical specimens, were selected on the basis that they had been initially classified as Gram-positive cocci during routine diagnostic procedures and were available for further characterization. Laboratory records were limited to the anatomical site of isolation and clinical context, without any patient-identifying information. Following primary isolation, all strains were preserved at −80 °C until further analysis. For phenotypic characterization, frozen stocks were subcultured on Blood Agar (Oxoid, Basingstoke, Hampshire, UK) and incubated at 37 °C for 24 h. Colonies displaying the typical creamy, opaque, golden-pigmented morphology were subjected to conventional biochemical identification. The identification workflow included Gram staining, catalase testing using 3% hydrogen peroxide, tube coagulase testing with rabbit plasma (Becton Dickinson, Franklin Lakes, NJ, USA), and culture on Mannitol Salt Agar (MSA) (Oxoid, Basingstoke, Hampshire, UK) to assess selective growth and mannitol fermentation [81]. All isolates displayed the characteristic biochemical profile of *S. aureus*, including Gram-positive cocci arranged in grape-like clusters, positive catalase and coagulase reactions, and growth with mannitol fermentation on MSA. SAT1 (traumatology wound), SAT2 (traumatology wound), and SAP1 (pediatric wound) were community isolates, whereas SAI1 (intensive care unit [ICU], cerebrospinal fluid [CSF]) and SAS1 (surgical wound) were hospital isolates.

### 4.2. Antimicrobial Susceptibility Testing

All *S. aureus* isolates were tested for antimicrobial susceptibility using the disk diffusion method (Kirby–Bauer) on Mueller–Hinton agar (Oxoid, Basingstoke, Hampshire, UK) with antibiotic disks (Oxoid, Basingstoke, Hampshire, UK) containing penicillin G (10 IU), cefoxitin (30 μg), azithromycin (15 μg), erythromycin (15 μg), clindamycin (2 μg), ciprofloxacin (5 μg), tetracycline (30 μg), gentamicin (10 μg), trimethoprim/sulfamethoxazole (1.25/23.75 μg), and linezolid (30 μg). *S. aureus* ATCC 25923 was used as the quality control strain and fell within the acceptable CLSI ranges on all test occasions. All susceptibility tests were performed in triplicate on independent occasions. Categories (susceptible, intermediate, and resistant) were interpreted according to the Clinical and Laboratory Standards Institute guidelines (CLSI M100, 36th edition, 2026) [82].

### 4.3. Whole-Genome Sequencing and Genomic Characteristics of S. aureus Strains

Extraction of DNA was carried out using the QIAamp UCP Pathogen Mini Kit (Qiagen Sciences INC, Germantown, MD, USA), while isolates were whole-genome-sequenced on an Illumina MiSeq platform (Illumina, San Diego, CA, USA). The quality of the reads was evaluated using FastQC v0.12 [83]; reads with a Q-score of 20 or lower were excluded through the application of Trimmomatic v0.39 [84]. De novo assembly was conducted using Unicycler v0.5.1 [85], configured to operate in bold mode; the K-mers were selected automatically, and the minimum contig size was set at 500 bp. QUAST v5.3.0. was used to evaluate the quality of each assembly [86], while contamination level and completeness were assessed with CheckM2 v1.1.0 [87]. Subsequently, the similarity percentage to the *S. aureus* reference (Accession code: CP088157.1) was determined using FastANI v1.34 (source code available at https://github.com/ParBLiSS/FastANI, accessed on 9 June 2026, [88]). The sequences were deposited in the Sequence Reads Archive (SRA) under BioProject PRJNA1503521, with BioSample ID 1503521. The sequence type was determined using MLST version 2.35.0 (source code available at https://github.com/tseemann/mlst, accessed 9 June 2026, [89]), based on seven consensus genes (*arcC*, *aroE*, *glpF*, *gmk*, *pta*, *tpi*, and *yqiL*). ABRicate version 1.4.0 [90] was employed to identify relevant genes, utilizing the NCBI AMRFinderPlus database v4.2 (available at https://github.com/ncbi/amr, accessed 10 June 2026, [91]) for resistance genes, the VFDB for virulence factors (available at https://www.mgc.ac.cn/VFs/, accessed 10 June 2026, [92]), and the PlasmidFinder v3.0 (available at https://cge.food.dtu.dk/services/PlasmidFinder/, accessed 10 June 2026, [93]) database for replicons.

Hits were considered positive at ≥90% nucleotide identity and ≥85% query coverage for resistance genes and plasmid replicons, and at ≥80% nucleotide identity and ≥80% query coverage for virulence genes. The more permissive thresholds for the latter were chosen to capture divergent allelic variants while minimizing spurious matches. Genes identified through the VFDB were grouped into functional categories based on the annotation provided by the database: adhesion and biofilm formation, toxins and secreted enzymes, iron acquisition, immune evasion, and invasion and secretion systems. SCCmecFinder v1.1.4 [94] was utilized to identify the *mec* chromosomal cassette using default parameters. Genome annotation was done with Bakta v1.12.0 [95]. Mobile genetic elements (MGEs) were screened using ABRicate v1.4.0 [90] with the TnCentral database [96] (accessed on 18 September 2026), and hits were considered positive at ≥90% nucleotide identity and ≥80% query coverage. Prophage sequences were identified using the PHASTEST web server [97] (https://bio.tools/phastest, accessed on 18 September 2026) with default parameters, which classifies detected regions as intact, questionable, or incomplete based on an internal scoring system. Because only short-read sequencing data were available, phage and transposon content was assessed as the presence or absence of signature genes and partial element sequences rather than complete element reconstruction, which requires long-read data. Finally, Proksee [98] was used to visualize the isolates, with resistance genes added using CARD.

### 4.4. Phylogenomic Analysis of S. aureus Isolates

To provide phylogenetic context for our isolates, we compiled a reference dataset comprising the 98 available *S. aureus* clinical genomes from Ecuador deposited in NCBI GenBank (accessed 02 June 2026, [99]). They were retrieved using the query *Staphylococcus aureus* [Organism] AND Ecuador [Country], and all met quality criteria of ≥95% completeness and ≤5% contamination as determined by CheckM2 v1.1.0 [87]. None had incomplete metadata, and no genome pairs reached the 100% ANI [88] threshold used to define duplicates in this study (Supplementary Materials: Genomes for comparative analyses; Supplementary Results: Raw Data). Genomes were then annotated and subjected to multiple alignments using PPanGGOLiN v2.3.0 [100]. Phylogenomic analysis was subsequently conducted using IQ-TREE v3.0.1 [101], incorporating the model finder and ultrafast bootstrap method. The genomic characteristics of the isolates were determined using the software packages described above. A second phylogenomic tree was developed using the same methodology, which included the eight most abundant sequence types (STs) along with the isolates from the current study.

To situate the Ecuadorian ST8, ST30, and ST72 isolates within their respective globally circulating lineages, reference genomes representing the major subclades of each sequence type were obtained from NCBI and were required to meet the same quality criteria previously mentioned (accessed on 18 September 2026, [99]) (Supplementary Materials, Genomes for comparative analyses). For ST8, five references were included: the classic North American epidemic clone USA300-NAE, the Latin American variant USA300-LV (CA12, Colombia), the older hospital-associated lineage USA500 historically proposed as a USA300 ancestor, an ancestral PVL+ MSSA from Trinidad, and a “Basal African” representative (Gabon) [28]. For ST30, four references were included: the historical PT80/81 pandemic MSSA clone G1-Pk, the Southwest Pacific (SWP) clone G2-Sg1-Yw, the “silent” contemporary MSSA lineage G2-Sg2-Pr, and the Brazilian MRSA subgroup G2-Sg3-Gn [30]. For ST72, four references were used, corresponding to the clades defined by Zhou et al. [43]: clade A, the earliest-diverging ST72 lineage (M0139); clade B, a highly virulent clade (SKLX56774); clade C, the Korean lineage (CN1); and clade D, the MSSA-only sublineage (3688STDY6124895). All reference genomes were required to meet the mentioned quality criteria, and were analyzed using the core-genome alignment and phylogenomic pipeline described above. A total of 51 genomes were included: 38 Ecuadorian (28 ST8, 8 ST30, and 2 ST72) and 13 reference genomes (5 ST8, 4 ST30, and 4 ST72).

ST8 isolates were screened for the arginine catabolic mobile element (ACME) and the copper and mercury resistance element (COMER). Protein sequences for ACME-associated genes (*arcA*, *arcB*, *arcC*, *arcD*, *arcR*, *opp-3A–E*, *kdpA–E*) and COMER-associated genes (*merR*, *merA*, *merB*, *copZ*, *mco*) were obtained from the USA300 and CA12 reference genomes, respectively, and queried against the two ST8 genomes using tblastn within NCBI BLAST+ v2.17.0+ [102]. Hits were considered positive at ≥90% amino acid identity and ≥80% query coverage. In addition, the core SNP distance between the two ST8 isolates was determined using Snippy v4.6.0 [103], using SAT2 as the reference. Subsequently, an investigation was conducted on ST2625 involving a search of the NCBI BioSample database for isolates belonging to this ST. Genomes were retrieved using the query *Staphylococcus aureus* [Organism] AND ST2625, and were required to meet the same quality parameters described above. For this analysis, de novo assembly was performed on 10 isolates available in NCBI (accessed 20 June 2026, [99]) and one isolate from the present study (Supplementary Materials: Genomes for comparative analyses) using Unicycler v0.5.1 [85]. For phylogenomic reconstruction, Snippy v4.6.0 [103] was employed to obtain core genome single-nucleotide polymorphisms (cgSNPs), using the *S. aureus* genome GCA_022494545 as a reference. Gubbins v3.4 [104] was then used to mitigate errors caused by recombination events, after which phylogenomic reconstruction via maximum likelihood was carried out with IQ-TREE v3.0.1 [101]. Genomic characteristics of the ST2625 isolates were assessed as already described. Phylogenetic trees were visualized and edited using iTOL v6 web platform [105]. Pairwise ANI values were calculated between the five study isolates and the Ecuadorian, Dutch ST2625, phylogenomic reference, and two type strain genomes NCTC 8325 (GCA_000013425.1) and ATCC 51811 (GCA_000164715.1). All comparisons were performed with FastANI v1.34 [88] run with default parameters.

## 5. Conclusions

This study provides additional genomic characterization of five clinical *S. aureus* isolates and illustrates the diversity of lineages represented in the analyzed Ecuadorian collection, including both commonly reported and less frequently described sequence types. Integration of phenotypic antimicrobial susceptibility testing with whole-genome sequencing showed generally concordant resistance profiles. Comparative genomic analysis identified variation among sequence types in antimicrobial resistance determinants, virulence-associated genes, and replicon content. Placement within globally circulating lineages showed that the ST8 isolates clustered with USA300-LV (CA12), the ST30 isolate with G2-Sg2-Pr, and the ST72 isolate with clade B, all with high ANI to Ecuadorian genomes of the same sequence type. The focused analysis of ST2625 identified limited genomic divergence and broadly similar resistance and virulence-associated profiles among the Ecuadorian and international genomes examined. However, the small number of study isolates and their origin from a single hospital limit the generalizability of these findings. Further genomic characterization of isolates from additional geographic regions and healthcare settings could help determine whether the patterns observed here extend to the wider Ecuadorian *S. aureus* population.

## Figures and Tables

**Table 1:** Antibiotic susceptibility test (Kirby–Bauer Method).

Antibiotic Class	Antibiotic Disks	Diameter of Inhibition (mm ± SD)				
		SAT1	SAT2	SAP1	SAI1	SAS1
β-lactams	Penicillin G (10 IU)	15.3 ± 0.6 (R)	12.0 ± 1.0 (R)	14.3 ± 1.2 (R)	12.3 ± 1.2 (R)	6.3 ± 0.6 (R)
	Cefoxitin (30 μg)	22.0 ± 1.0 (S)	18.0 ± 1.0 (R)	17.3 ± 0.6 (R)	16.7 ± 0.6 (R)	6.3 ± 1.2 (R)
Macrolide	Azithromycin (15 μg)	6.3 ± 0.6 (R)	25.2 ± 0.8 (S)	24.0 ± 1.2 (S)	6.0 ± 1.0 (R)	22.7 ± 2.1 (S)
	Erythromycin (15 μg)	8.3 ± 0.6 (R)	28.3 ± 2.1 (S)	28.7 ± 0.6 (S)	9.3 ± 0.6 (R)	26.3 ± 1.2 (S)
Lincosamide	Clindamycin (2 μg)	6.3 ± 2.3 (R)	28.3 ± 2.1 (S)	27.7 ± 0.6 (S)	29.3 ± 0.6 (S)	25.3 ± 0.6 (S)
Fluoroquinolone	Ciprofloxacin (5 μg)	27.3 ± 1.2 (S)	24.3 ± 1.5 (S)	23.3 ± 0.6 (S)	29.7 ± 0.6 (S)	25.7 ± 1.2 (S)
Tetracycline	Tetracycline (30 μg)	23.7 ± 1.5 (S)	29.0 ± 1.0 (S)	29.7 ± 0.6 (S)	13.3 ± 0.6 (R)	12.3 ± 0.6 (R)
Aminoglycoside	Gentamicin (10 μg)	16.7 ± 1.2 (S)	23.3 ± 1.2 (S)	22.0 ± 1.0 (S)	10.3 ± 0.6 (R)	11.0 ± 1.0 (R)
Antifolates	Trimethoprim/Sulfamethoxazole (1.25/23.75 μg)	16.3 ± 0.6 (S)	24.0 ± 1.7 (S)	22.7 ± 1.2 (S)	26.7 ± 3.2 (S)	16.3 ± 0.6 (S)
Oxazolidinone	Linezolid (30 μg)	29.0 ± 1.0 (S)	30.0 ± 1.7 (S)	29.7 ± 0.6 (S)	30.0 ± 2.0 (S)	30.3 ± 1.6 (S)

R: resistant. S: susceptible.

**Table 2:** Genomic and epidemiological features of *S. aureus* isolates.

Features	Details				
	SAT1	SAT2	SAP1	SAI1	SAS1
Source	Clinical (community, traumatology wound)	Clinical (community, traumatology wound)	Clinical (community, pediatric wound)	Clinical (CSF sample from ICU)	Clinical (surgery wound)
Genome size (bp)	2,851,947	2,844,422	2,839,640	2,807,961	2,797,170
No. of CDS	2695	2653	2638	2598	2587
GC content	32.73%	32.65%	32.66%	32.70%	32.73%
MLST	30	8	8	72	2625
ARGs	- Aminoglycosides: *ant (9)-Ia* - β-lactams: *blaI*, *blaR1*, *blaZ* - Macrolides/Lincosamides: *erm (A)* - Fosfomycin: *fosB*	- β-lactams: *blaI*, *blaR1*, *blaZ*, *mecA* - Folate Antagonists: *dfrG* - Fosfomycin: *fosB*	- β-lactams: *blaI*, *blaR1*, *blaZ*, *mecA* - Folate Antagonists: *dfrG* - Fosfomycin: *fosB*	- Aminoglycosides: *ant (4′)-Ia*, *aph (3′)-IIIa* - β-lactams: *blaI*, *blaR1, blaZ*, *mecA* - Macrolides: *mph (C)*, *msr (A)* - Tetracycline: *tet(K)* - Folate Antagonists: *dfrS1* - Fosfomycin: *fosB* - Bleomycin: *ble0* - Streptothricin: *sat4*	- Aminoglycosides: *aph (2″)-Ii* - β-lactams: *blaI*, *blaR1*, *blaZ*, *mecA* - Tetracycline: *tet (L)*, *tet (M)* - Fosfomycin: *fosB*
*SCCmec* type	n.d.	n.d. (*mecA*, *ccrA2*, *ccrB2*, *IS1272*, *mecR1*; untypeable)	IV (*mecA*, *ccrA2*, *ccrB2*, *IS431*, *IS1272*, *mecR1*; subtype ambiguous)	IV (*ccrA2*, *ccrB2*, *IS431*, *IS1272*, *mecR1*; hybrid/mosaic elements: IVa, IVn)	IV (*ccrA2*, *ccrB2*, *IS431*, *IS1272*, *mecR1*; hybrid/mosaic elements: IVa, IVn)
Replicons	rep19_7_repA (SAP019A)	rep20_3_rep (pTW20); rep7c_1_rep (MSSA476)	rep20_3_rep (pTW20); rep7c_1_rep (MSSA476)	rep7a_16_repC (Cassette); rep7c_1_rep (MSSA476)	rep20_3_rep (pTW20); rep21_9_rep (pKH12); repUS43_1_CDS12738 (DOp1)
MGEs	ISSau1; ISSau2; ISSau3; ISSau5; ISSau6; Tn554	IS1181; ISSau5; ISSau6; ISSep	IS1181, IS431mec; ISSau5; ISSau6	IS1181; IS257R2; ISSep1; ISSep3	IS1181; IS431mec; ISSau1
Prophages	*S. aureus* phi2958PVL; *S. aureus* PT1028	*S. aureus* phage 11; *S. aureus* phi2958PVL	*S. aureus* phage 11; *S. aureus* MC/09/04/R1988	*S. aureus* phage 11; *S. aureus* SA345ruMSSAST8	*S. aureus* phage 69; *S. aureus* phiN315

CSF: cerebrospinal fluid; ICU: intensive care unit; ARG: antimicrobial resistance genes; n.d.: not detected.

## Data Availability

The genomic sequences generated for this study have been deposited in the Sequence Reads Archive (SRA) under BioProject PRJNA1503521, with BioSample ID 1503521. The publicly available genome sequences of *S. aureus* ST2625 from Ecuador and worldwide, which were analyzed in this study, can be found in the NCBI database (https://www.ncbi.nlm.nih.gov/) as cited in the text. The original contributions presented in this study are included in the article/Supplementary Materials.

## Supplementary Materials

The following supporting information can be downloaded at https://www.mdpi.com/article/10.3390/ijms27209135/s1.
